# Plasma Neurofilament Light Chain and TNF‐α Correlate with Motor Features in Isolated REM Sleep Behavior Disorder

**DOI:** 10.1002/mdc3.70210

**Published:** 2025-07-14

**Authors:** Brook Huxford, Cristina Simonet, Laura Pérez‐Carbonell, Guy Leschziner, Anette Schrag, Ruth Dobson, Alastair J. Noyce

**Affiliations:** ^1^ Centre for Preventive Neurology Charterhouse Square, Queen Mary University of London London UK; ^2^ Sleep Disorders Centre Guy's and St Thomas' NHS Foundation Trust London UK; ^3^ Department of Clinical and Movement Neurosciences UCL Institute of Neurology London UK; ^4^ Royal London Hospital, Barts Health NHS Trust London UK

**Keywords:** RBD, neurofilament, inflammation, biomarkers, Parkinson's

## Abstract

**Background:**

Isolated RBD (iRBD) is a prominent early marker of Parkinson's disease and related disorders.

**Objectives:**

Evaluate biomarkers of inflammation and axonal damage people with iRBD for disease progression.

**Methods:**

Plasma samples from 27 video‐polysomnography confirmed iRBD patients and 25 controls were analyzed for inflammatory biomarkers (CRP, IL‐6, TNF‐α, IFN‐γ, IL‐1β) and NfL using the Meso Scale Discovery platform.

**Results:**

People with iRBD had elevated plasma TNF‐α and IL‐6 (*p* = 0.013 and *p* = 0.051). In the iRBD group, plasma NfL was associated with objective motor tests: Timed Up and Go (*R* ^2^ = 0.292, *p* = 0.027) and 10‐m walk (*R* ^2^ = 0.355, *p* = 0.034) and plasma TNF‐α was associated with the Timed Up and Go test (*R* ^2^ = 0.277, *p* = 0.025).

**Conclusions:**

We report evidence of peripheral inflammation in early synuclein‐related disease processes, and association of plasma NfL and TNF‐α with motor dysfunction in iRBD patients, suggesting possible association with increased risk of phenoconversion.

The overt clinical features of Parkinson's disease (PD) manifest relatively late in the disease process, therefore biological markers are required to enable diagnosis of PD at earlier stages.^1^ Isolated rapid eye movement (REM) sleep behavior disorder (iRBD) is a parasomnia characterized by dream enactment episodes, the diagnosis of which requires video‐polysomnography (v‐PSG). Recent data show a 73.5% conversion rate from iRBD to neurodegenerative disease after 12‐year follow‐up, with equal proportions of people converting to either PD or DLB.^2^, ^3^, ^4^, ^5^, ^6^, ^7^ These data are substantiated by synuclein seed amplification assay (SAA) results, with pathogenic forms of alpha‐synuclein present in the cerebrospinal fluid (CSF) of 90–100% of patients with iRBD.^8^, ^9^, ^10^

Despite progress in the field, individual determinants of phenoconversion are still relatively unknown. There is growing evidence that blood NfL correlates with both motor and cognitive symptom severity in both PD and DLB.^11^, ^12^, ^13^, ^14^, ^15^, ^16^, ^17^, ^18^, ^19^, ^20^ There is also increasing evidence for the role of NfL in iRBD; intraindividual increase of NfL was observed in RBD patients progressing to PD,^21^ and subsequent studies have shown that baseline NfL is predictive of disease progression and phenoconversion in iRBD patients.^22^, ^23^ Inflammation has also been implicated as a feature of early disease in iRBD.^24^, ^25^ Cross‐sectionally, two studies have published negative results, whilst one study has reported elevated levels of plasma TNF‐α in iRBD patients compared to controls.^26^, ^27^, ^28^ Inflammatory cytokines may also serve as severity biomarkers in the earliest stages of PD; elevated plasma TNF‐α has been associated with risk of phenoconversion of iRBD patients.^26^, ^27^ However, it is unclear if pro‐inflammatory cytokines and NfL in the blood can serve as markers of disease severity.

## Methods

### Participants

Patients with iRBD, confirmed by v‐PSG, were recruited through the sleep clinic at Guy's and St Thomas’ NHS Foundation Trust and enrolled into the PREDICT‐PD study. Age‐ and sex‐matched controls were also recruited from PREDICT‐PD. Patients with iRBD with suspected PD at the time of assessment were excluded from the study and long‐term follow up data were not available.

### Clinical Assessments

Full details of clinical assessments have been previously published.^29^ For this study, we used the MDS Unified PD Rating Scale Part 3 (MDS‐UPDRS III), the Timed Up and Go (TUG) test (the mean of three attempts), the 10‐m walk test (10MWT), and the Montreal Cognitive Assessment (MoCA).

### Blood Sample Collection and Analysis

Full details of blood sample collection and processing can be seen in the supplementary materials. Venous K_2_EDTA blood was centrifuged at 2000 g for 15 min, plasma supernatant was then re‐centrifuged at 2000 g for 15 min to produce platelet‐depleted plasma. Plasma was stored at −80°C and had undergone only one freeze–thaw cycle. Plasma samples were analyzed in at least duplicate using Meso Scale Discovery (MSD) electrochemiluminescence (ECL) immunoassay kits (MSD R‐Plex NfL kit for NfL, MSD V‐Plex pro‐inflammatory Panel I kit for IL‐6, TNF‐α, IFN‐ɣ, and IL‐1β, and the V‐Plex CRP kit for CRP). Quality control analyses of MSD data can be seen in the supplementary materials (Tables S1–S6).

### Statistical Analysis

Full details of statistical analyses are in the supplementary e‐Methods. For case–control comparisons for biomarker data, both raw and log‐transformed data were tested for normality; the *t*‐test was used if data were normally distributed and the Mann Whitney *U*‐Test used for non‐normally distributed data, with a nominal significance of 5%. Age‐ and sex‐ adjustment was carried out using a linear model.

Multiple linear regression was used to assess the association between log plasma biomarker levels of NfL, TNF‐α, and IL‐6 and clinical data in both the iRBD and control groups; biomarkers were chosen due to evidence from case–control comparisons. Data were reported as *R* ^2^ and *p* values after adjustment for age and sex. Age‐ and sex‐ adjusted linear regression slopes were compared between the iRBD and control groups with a nominal significance of 5%.

Data analyses were carried out using Meso Scale DISCOVERY WORKBENCH Desktop Analysis Software, R Studio (version 2024.12.1) and Graph Pad Prism (version 10.4.0 (621)).

## Results

### Demographics

Of the participants eligible for the study, 27 iRBD patients and 25 controls provided blood samples (Table S7). The groups were matched by age (average age: RBD: 68.4 years, controls: 69.6 years) and sex, with a higher proportion of males than females in both groups (iRBD: 81.5% male, controls: 84% male).

### Clinical Outcomes

Significantly worse MoCA and MDS‐UPDRS III scores were seen in iRBD patients versus controls (MoCA: iRBD: 26.4 ± 2.90 versus controls: 28.6 ± 1.19; *p* = 0.001), (MDS‐UPDRS III: iRBD: 6.85 ± 4.70 versus controls: 3.16 ± 2.41; *p* < 0.001) (Table S7, Fig. S1). No differences were observed between groups for the 10MWT or the TUG test (Table S7, Fig. S1).

### Plasma Findings

Plasma levels of pro‐inflammatory cytokines TNF‐α and IL‐6 were elevated in the iRBD group compared to controls (TNF‐α: iRBD: 1.70 ± 0.35 pg/mL versus controls: 1.45 ± 0.33 pg/mL; *p* = 0.013), (IL‐6: iRBD: 1.50 ± 1.35 pg/mL vs. controls: 0.99 ± 0.45 pg/mL) (Table 1, Fig. S2). There were no statistically significant differences in levels of plasma NfL, CRP, IFN‐ɣ or IL‐1β between individuals with iRBD and controls. Age and sex adjusted models are also reported for plasma biomarker differences in the supplementary material (Fig. S3, Table S8).

**TABLE 1:** Plasma biomarker levels for iRBD patients and healthy controls (HC).

	iRBD	HC	*p*‐value
[Plasma TNF‐α], pg/mL Mean (SD)	1.70 (0.352) *n =* 27	1.45 (0.326) *n =* 23	*0.013_*T*_
[Plasma IL‐6], pg/mL Mean (SD)	1.50 (1.35) *n =* 24	0.987 (0.451) *n =* 22	0.051_MW_
[Plasma NfL], pg/mL Mean (SD)	80.3 (24.7) *n =* 27	74.0 (38.7) *n =* 25	0.085_MW_
[Plasma IL‐1β], pg/mL Mean (SD)	0.181 (0.158) *n =* 16	0.190 (0.144) *n =* 17	0.533_MW_
[Plasma IFN‐ɣ], pg/mL Mean (SD)	7.40 (6.96) *n =* 23	6.27 (4.22) *n =* 24	0.597_*T*(log)_
[Plasma CRP], mg/dL Mean (SD)	0.225 (0.258) *n =* 27	0.137 (0.093) *n =* 25	0.485_*T*(log)_

*Note*: Plasma biomarker levels are given as mean levels and standard deviations (SD).

Abbreviations: MW, Mann Whitney *U*‐Test; *n*, number of participants; *T*, *T*‐test.

* *p* < 0.05.

### Correlations With Clinical Outcomes

**Figure 1:**
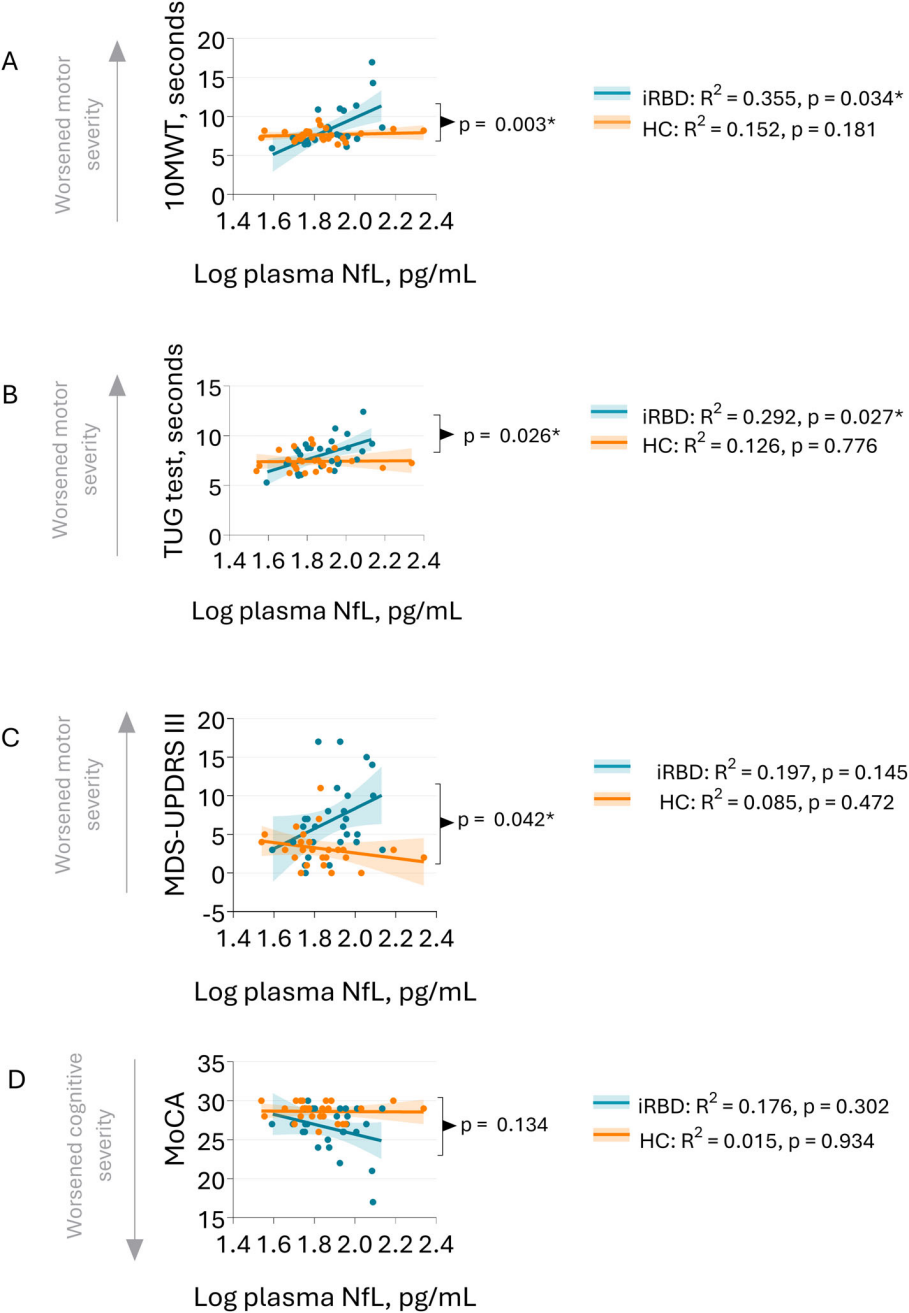
Linear regression of log plasma NfL with clinical markers. (A) 10‐m walk test (10MWT), (B) Timed Up and Go (TUG) test, (C) MDS‐UPDRS III, (D) MoCA. Reported *p*‐values are adjusted for age and sex. Biomarker data is log (base 10) transformed.

Tables S9 and S10 include the results of multiple linear regression of plasma and log plasma NfL, TNF‐α, and IL‐6, and clinical severity, respectively, adjusting for age and sex. In the iRBD group, associations were observed between log plasma NfL and objective outcome motor measures: TUG test (*R* ^2^ = 0.292, *p* = 0.027), and the 10MWT (*R* ^2^ = 0.355, *p* = 0.034) (Fig. 1, Table S10). There was, however, no correlation for the iRBD group between log plasma NfL and MDS‐UPDRS III (*R* ^2^ = 0.197, *p* = 0.145), or between plasma NfL and MoCA (*R* ^2^ = 0.176, *p* = 0.302) (Fig. 1, Table S10). Further analysis revealed that regression slopes were significantly different between iRBD and controls for log plasma NfL and the 10MWT, TUG test, and MDS‐UPDRS III (*p* = 0.003, *p* = 0.026, *p* = 0.042, respectively).

In the iRBD group, an association was also observed between log plasma TNF‐α and the TUG test (*R* ^2^ = 0.277, *p* = 0.025) (Fig. S4, Table S10), however, no correlation was observed between log plasma TNF‐α and any other clinical markers, nor between plasma IL‐6 and any clinical markers (Fig. S5).

For the control group, except for an association between log plasma IL‐6 and MDS‐UPDRS III (*R* ^2^ = 0.319, *p* = 0.026) (Fig. S5), no other associations were observed between either plasma NfL, TNF‐α, or IL‐6 and any clinical assessments.

## Discussion

This cross‐sectional study demonstrates elevated levels of TNF‐α and IL‐6 in people with iRBD compared to controls. Additionally, we report for the first time that in people with iRBD, plasma levels of NfL and TNF‐α correlate with objective markers of motor symptom severity, but no correlation was found between plasma biomarkers and cognitive symptom severity.

Previous research has shown TNF‐α to be elevated in the blood, CSF, and brains of PD patients,^30^, ^31^, ^32^, ^33^, ^34^ and data suggests TNF‐α plays a role in nigral degeneration.^35^ Data on blood levels of TNF‐α in iRBD patients have been inconsistent, but elevated plasma TNF‐α in people with iRBD compared to controls has been previously reported.^26^ Our data supports such evidence that inflammation may play a role early on in the PD process, as we observed raised levels of pro‐inflammatory cytokines TNF‐α and IL‐6 in iRBD patients compared to controls.^36^, ^37^ In the iRBD group, we also report a correlation between plasma TNF‐α and the TUG test, after adjustment for age and sex, adding further evidence to the role of inflammation in early PD and disease prognosis. Several studies have also shown inflammatory markers to be associated with clinical symptom severity in PD patients^38^, ^39^ and people with iRBD.^26^, ^27^

We report correlations between clinical motor severity and plasma NfL in the iRBD patient group, which was not seen in the control group. The value of blood NfL as a motor progression biomarker has been previously reported in PD and DLB patients.^11^, ^12^, ^13^, ^14^, ^15^ In people with iRBD, intra‐individual increase of NfL levels has been reported in individuals converting to manifest motor stage^21^ and several studies have also shown that baseline NfL is predictive of disease progression and phenoconversion in people with iRBD.^22^, ^23^ Whilst our results align with existing literature showing no correlation between plasma NfL and MDS‐UPDRS III in individuals iRBD,^22^ we did see a difference in the regression slopes between iRBD and controls for MDS‐UPDRS III (*p* = 0.042). Through the use of objective, quantitative motor tests (capable of detecting subtle changes in motor severity),^29^ we observed a correlation between plasma NfL and the 10MWT (*R* ^2^ = 0.355, *p* = 0.034) which was further strengthened by using the TUG test (*R* ^2^ = 0.292, *p* = 0.027). Notably, however, no significant difference in plasma NfL was observed between the iRBD and control groups cross‐sectionally, likely due to the early stage of the disease, where elevation of plasma NfL levels in the iRBD group are minimal. These objective tools of motor symptom severity, however, may be sensitive enough to detect subtle motor changes during the earliest stages of PD, and therefore elucidate correlations with plasma NfL. To the best of our knowledge, our results are the first to show cross‐sectionally that plasma NfL may be sensitive enough to detect early signs of motor impairment (using the TUG test and the 10MWT) in people with iRBD.

No correlations were seen between plasma NfL and cognitive outcomes in the iRBD group. While previous studies have shown the value of plasma NfL as a cognitive severity biomarker in PD and DLB patients,^13^, ^15^, ^16^, ^17^, ^18^ the evidence in iRBD is limited, with only one study showing that a higher baseline level of plasma NfL in iRBD patients is associated with a higher risk for cognitive progression.^22^ Our negative findings might suggest that, at this very early, prodromal, stage of neurodegenerative disease, plasma NfL does not serve a role in predicting cognitive decline. Alternatively, the MoCA assessment may not be sensitive enough to capture cognitive decline at this early stage, and a more extensive cognitive assessment may be required to find a relationship between plasma NfL and cognitive symptoms. Additionally, as people with iRBD are equally likely to develop PD or DLB, and in general NfL levels are higher in DLB than in PD,^40^ this may introduce variability into the data.

A limitation of the present study is that despite the statistical differences observed, there is significant overlap in biomarker levels between the iRBD and control group, which may limit the suitability of these markers for use in clinic. The sample size and cross‐sectional design are also limitations of the current study. Larger numbers of people with iRBD and longitudinal disease outcome data are needed for iRBD biomarker studies, to confirm the value of plasma NfL or inflammatory markers as disease progression biomarkers in well‐stratified iRBD cohorts.

Our study identified elevated plasma TNF‐α and IL‐6 in people with iRBD compared with controls. In people with iRBD, a correlation was also observed between plasma biomarkers (NfL, TNF‐α) and motor severity. The biomarkers analysed in this study help to shed light on the biology of iRBD and may serve as progression markers, however, they do not directly measure synucleinopathy (or other neuropathological changes) in early PD. Likely, the utility of biomarkers in iRBD will be a combination of a synuclein‐based marker for diagnosis and blood NfL or inflammatory biomarkers, as shown in this study, for prediction and prognosis.

## Author Roles

(1) Research project: A. Conception, B. Organization, C. Execution; (2) Statistical Analysis: A. Design, B. Execution, C. Review and Critique; (3) Manuscript Preparation: A. Writing of the first draft, B. Review and Critique.

B.H.: 1A, 1B, 1C, 2A, 2B, 3A

C.S.:1B, 1C, 3B

L.P.‐C.: 1B, 1C, 3B

G.L.: 1C, 3B

A.S.: 1A, 2C, 3B

R.D.: 1A, 2C, 3B

A.N.: 1A, 2C, 3B

## Disclosure

**Ethical Compliance Statement:** This study was approved by the Research Ethics Committee (REC) (PREDICT‐PD, REC Ref: 10/H0716/85) and patient consent was recorded digitally. We confirm that we have read the Journal's position on issues involved in ethical publication and affirm that this work is consistent with those guidelines.

**Funding Sources and Conflict of Interest:** No specific funding was received for this work. The authors declare that there are no conflicts of interest relevant to this work. The PREDICT‐PD study is funded by Parkinson's United Kingdom. The Centre for Preventive Neurology Unit is funded by the Barts Charity.

**Financial Disclosures for the Previous 12 Months:** CS received research funding from the PPMI Early Investigators Program. AS receives research funding or support from University College London, National Institute of Health (NIHR), National Institute for Health Research, UCLH Biomedical Research Centre, the International Parkinson and Movement Disorder Society (IPMDS), the European Commission, Parkinson's UK. AS receives honoraria for consultancy from Biogen, Abbvie, Bial, Otsuka. RD received research support from Merck, Biogen and Celgene; consultancy fees from F. Hoffmann‐La Roche Ltd, Novartis, Sandoz and Biogen (all payments made were institutional and used to support research/educational activities); honoraria from Biogen, F. Hoffmann‐La Roche Ltd, Sanofi‐Genzyme, Merck, Novartis, Janssen and Esai. AJN received grants from Parkinson's UK, Barts Charity, Cure Parkinson's, National Institute for Health and Care Research, Innovate UK, Solvemed, the Medical College of Saint Bartholomew's Hospital Trust, Alchemab, Aligning Science Across Parkinson's Global Parkinson's Genetics Program (ASAP‐GP2) and the Michael J. Fox Foundation. AJN receives consultancy and personal fees from AstraZeneca, AbbVie, Profile, Bial, Charco Neurotech, Alchemab, Sosei Heptares, Umedeor and Britannia. AJN has share options in Umedeor. AJN is Associate Editor for the Journal of Parkinson's Disease. The authors declare that there are no additional disclosures to report.

## Supporting information


**Figure S1.** Comparison of clinical scores in people with iRBD (*n* = 27) and healthy controls (HCs) (*n* = 25). (A) 10 m Walk Test (10MWT), (B) Timed Up and Go Test (TUG), (C) Movement Disorders Society Unified Parkinson's Disease Rating Scale Part 3 (MDS‐UPDRS III), (D) Montreal Cognitive Assessment (MoCA). HC, healthy control; iRBD, isolated REM sleep behavior disorder. *p*‐values calculated using the Mann–Whitney *U* test. *p < 0.05.

**Figure S2.** Plasma biomarker levels in iRBD patients vs controls. (Top) Mean log plasma levels of NfL, IL‐6, and TNF‐α in iRBD patients compared to HCs. (Bottom) Mean log plasma levels of CRP, IFN‐ɣ, and IL‐1β in iRBD patients compared to HCs. *p* values are calculated using the *T*‐test/Mann Whitney *U* Test. *p < 0.05 (un‐adjusted *p*‐value).

**Figure S3.** Quantile‐quantile (Q‐Q) plots for log plasma biomarkers. Q‐Q plots for log (base 10) transformed biomarkers are displayed for log (10) plasma TNF‐α, log (10) plasma IL‐6, log (10) NfL, log (10) plasma IL‐1β, log (10) plasma IFN‐ɣ, and log (10) plasma CRP. Q‐Q plots were visually inspected for normality to assess the suitability of the linear model for age and sex adjustment in the case–control biomarker analysis.

**Figure S4.** Linear regression of plasma TNF‐α with clinical markers. (A) 10 m walk test (10MWT), (B) Timed Up and Go (TUG) test, (C) MDS‐UPDRS III, and (D) MoCA. Reported *p*‐values are adjusted for age and sex.

**Figure S5.** Linear regression of plasma IL‐6 with clinical markers. (A) 10 m walk test (10MWT), (B) Timed Up and Go (TUG) test, (C) MDS‐UPDRS III, and (D) MoCA. Reported *p*‐values are adjusted for age and sex.

**TABLE S1.** Intra‐plate NfL assay characteristics. For each NfL plate, the lower limit of detection (LLOD) is reported, along with the average %Coefficient of Variation (CV) of all duplicate plasma samples, the number of plasma samples that fell outside the detection range, and the number of plasma samples with %CVs greater than 25%
**TABLE S2.** Inter‐plate NfL control sample assay characteristics. Control samples were chosen as low, medium, and high NfL plasma samples from first plate run. Reported for the three controls are: the average control NfL value across plates 2–4, the inter‐plate control %Coefficient of Variation (CV) across all four plates, the value of plasma NfL for each control from first plate run, and the average control % recovery for plates 2–4
**TABLE S3.** Intra‐plate CRP assay characteristics. For each CRP plate, the lower limit of detection (LLOD) is reported, along with the average %Coefficient of Variation (CV) of all duplicate plasma samples, the number of plasma samples that fell outside the detection range, and the number of plasma samples with %CVs greater than 25%
**TABLE S4.** Inter‐plate CRP control sample assay characteristics. Control samples were chosen as low, medium, and high CRP plasma samples from plate 1. Reported for the three controls are: the average control CRP value for plate 2, the inter‐plate control %Coefficient of Variation (CV) across the two plates, the value of plasma CRP for each control from plate 1, and the average control % recovery for plate 2. *Average of CRP control samples from plate 2; plate 1 was used to determine plasma CRP concentration of control samples and for calculation of % recovery
**TABLE S5.** Intra‐plate pro‐inflammatory cytokines (IFN‐ɣ, IL‐1β, IL‐6, and TNF‐α) assay characteristics. For each plate and cytokine, the lower limit of detection is reported, along with the average %Coefficient of Variation (CV) for all duplicate plasma samples run, the number of plasma samples that fell outside the detection range of the assay, and the number of plasma samples with %CVs greater than the cut‐off (25%). *Plasma samples run in singlicate
**TABLE S6.** Inter‐plate pro‐inflammatory cytokines (IFN‐ɣ, IL‐1β, IL‐6, and TNF‐α) control sample assay characteristics. For each control sample supplied by Meso Scale Discovery (MSD), the average concentration is reported across five plates, the inter‐plate control %coefficient of variation (CV) is reported, the expected control concentration from MSD is reported, along with the average control % recovery for the five plates
**TABLE S7.** Demographics and clinical data for iRBD patients (*n* = 27) and controls (*n* = 25) who were eligible for the study and provided a blood sample. All data are presented as mean and standard deviation, apart from gender, which is presented as the number of males as a percentage of each group. Pearson's chi squared test was used to compare gender between the two groups, and the Mann–Whitney *U* Test to compare between groups for all other data, with a significance of 5%. *p < 0.05. SD, standard deviation
**TABLE S8.** Plasma biomarker levels for iRBD patients and healthy controls (HC) adjusted for age and sex. Plasma biomarker levels are given as mean levels and standard deviations (SD). *p*‐values are given (A) un‐adjusted using *T*‐tests or Mann–Whitney *U* Test as in the main manuscript, (B) un‐adjusted using a linear model, and (C) adjusted for age and sex using a linear model. Effect size using Cohen's d is reported. *n* = number of participants, MW, Mann Whitney *U*‐Test; *T*, *T*‐test, *p < 0.05
**TABLE S9.** Correlation analyses of plasma NfL, TNF‐α, and IL‐6 with clinical markers. Clinical markers analyzed were 10‐m walk test (10MWT), Timed Up and Go (TUG) test, MDS‐UPDRS III, and MoCA. Data are raw (not log transformed). Reported *p*‐values are adjusted for age and sex. *p < 0.05
**TABLE S10.** Correlation analyses of log plasma NfL, TNF‐α, and IL‐6 with clinical markers. Clinical markers analyzed were 10‐m walk test (10MWT), Timed Up and Go (TUG) test, MDS‐UPDRS III, and MoCA. Plasma biomarker data are log transformed. Reported *p*‐values are adjusted for age and sex. *p < 0.05

## Data Availability

The data that support the findings of this study are available from the corresponding author upon reasonable request.
