# Supplementary figures and images for: Plasma Neurofilament Light Chain and TNF‐α Correlate with Motor Features in Isolated REM Sleep Behavior Disorder
Source: Mov Disord Clin Pract. 2025 Jul 14;12(11):1961–7. doi: 10.1002/mdc3.70210 (PMC12625127; doi:10.1002/mdc3.70210)

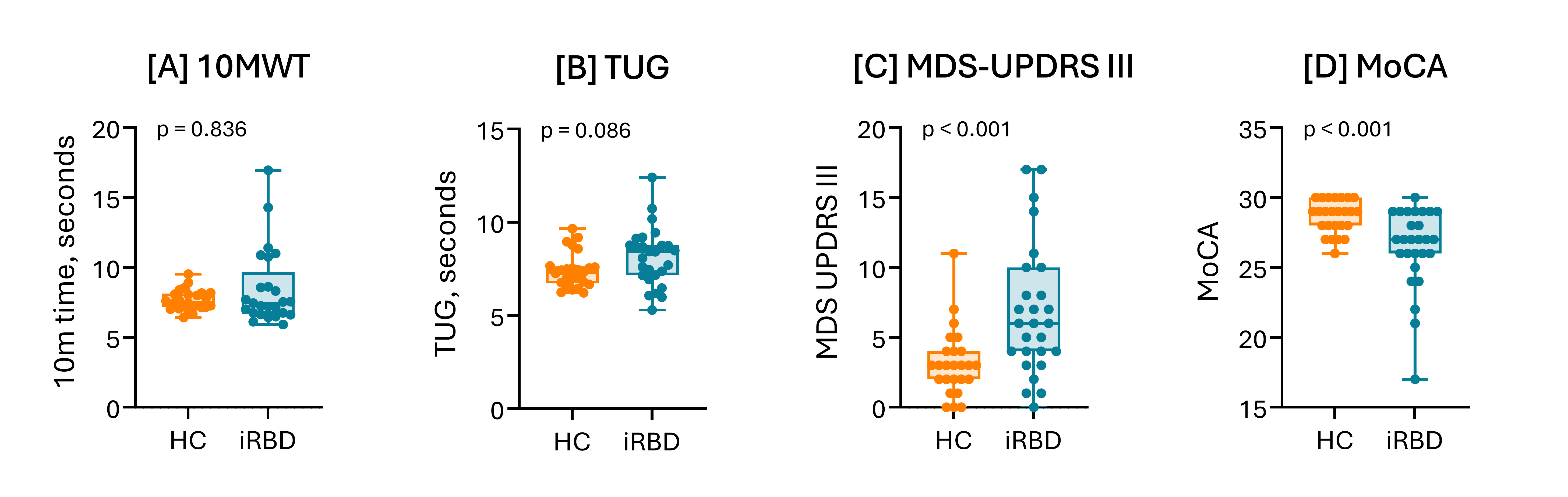

Supplement: Supplementary file 1 — Figure S1. Comparison of clinical scores in people with iRBD (n = 27) and healthy controls (HCs) (n = 25). (A) 10 m Walk Test (10MWT), (B) Timed Up and Go Test (TUG), (C) Movement Disorders Society Unified Parkinson's Disease Rating Scale Part 3 (MDS‐UPDRS III), (D) Montreal Cognitive Assessment (MoCA). HC, healthy control; iRBD, isolated REM sleep behavior disorder. p‐values calculated using the Mann–Whitney U test. *p < 0.05. [file MDC3-12-1961-s001.png]

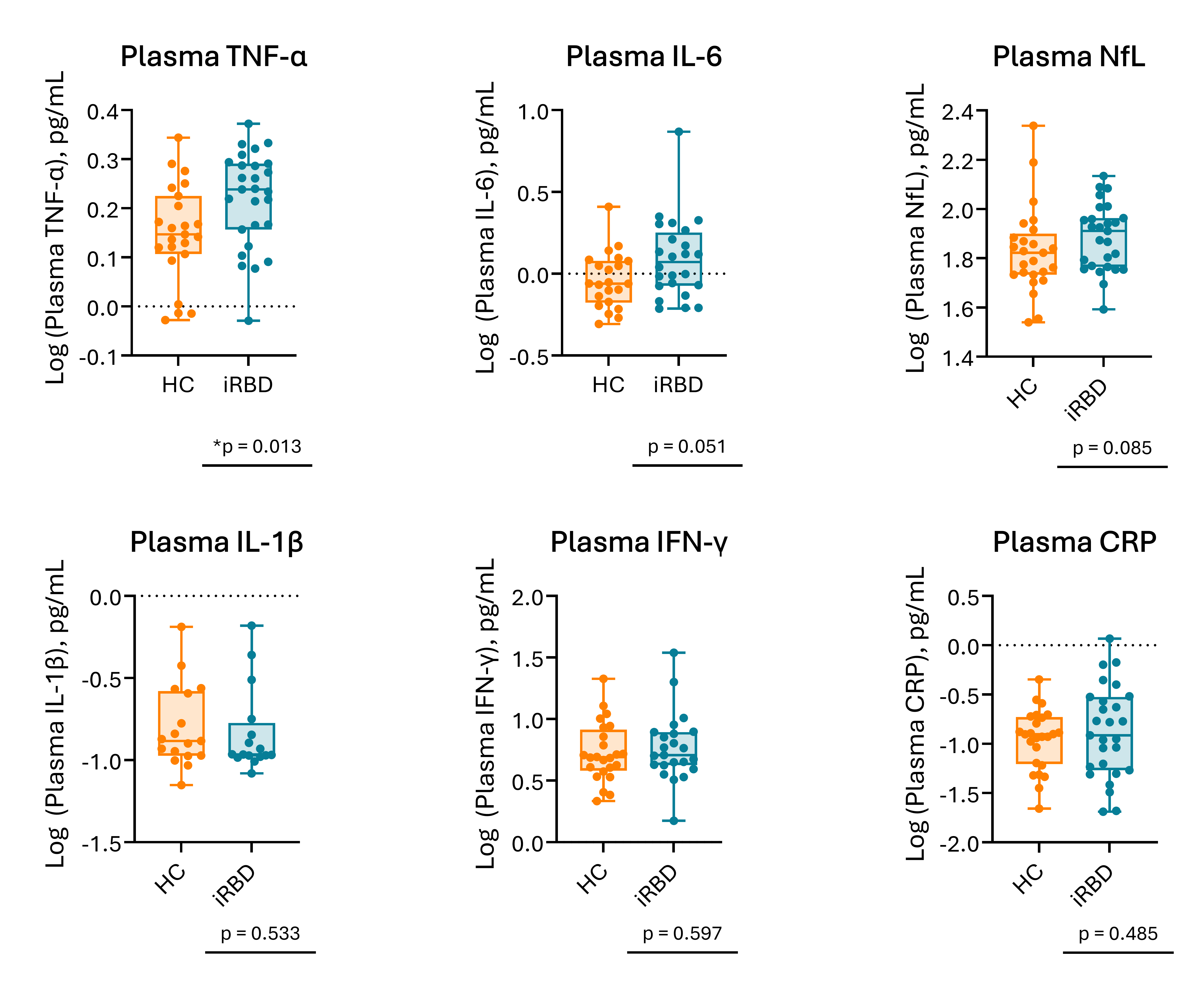

Supplement: Supplementary file 2 — Figure S2. Plasma biomarker levels in iRBD patients vs controls. (Top) Mean log plasma levels of NfL, IL‐6, and TNF‐α in iRBD patients compared to HCs. (Bottom) Mean log plasma levels of CRP, IFN‐ɣ, and IL‐1β in iRBD patients compared to HCs. p values are calculated using the T‐test/Mann Whitney U Test. *p < 0.05 (un‐adjusted p‐value). [file MDC3-12-1961-s006.png]

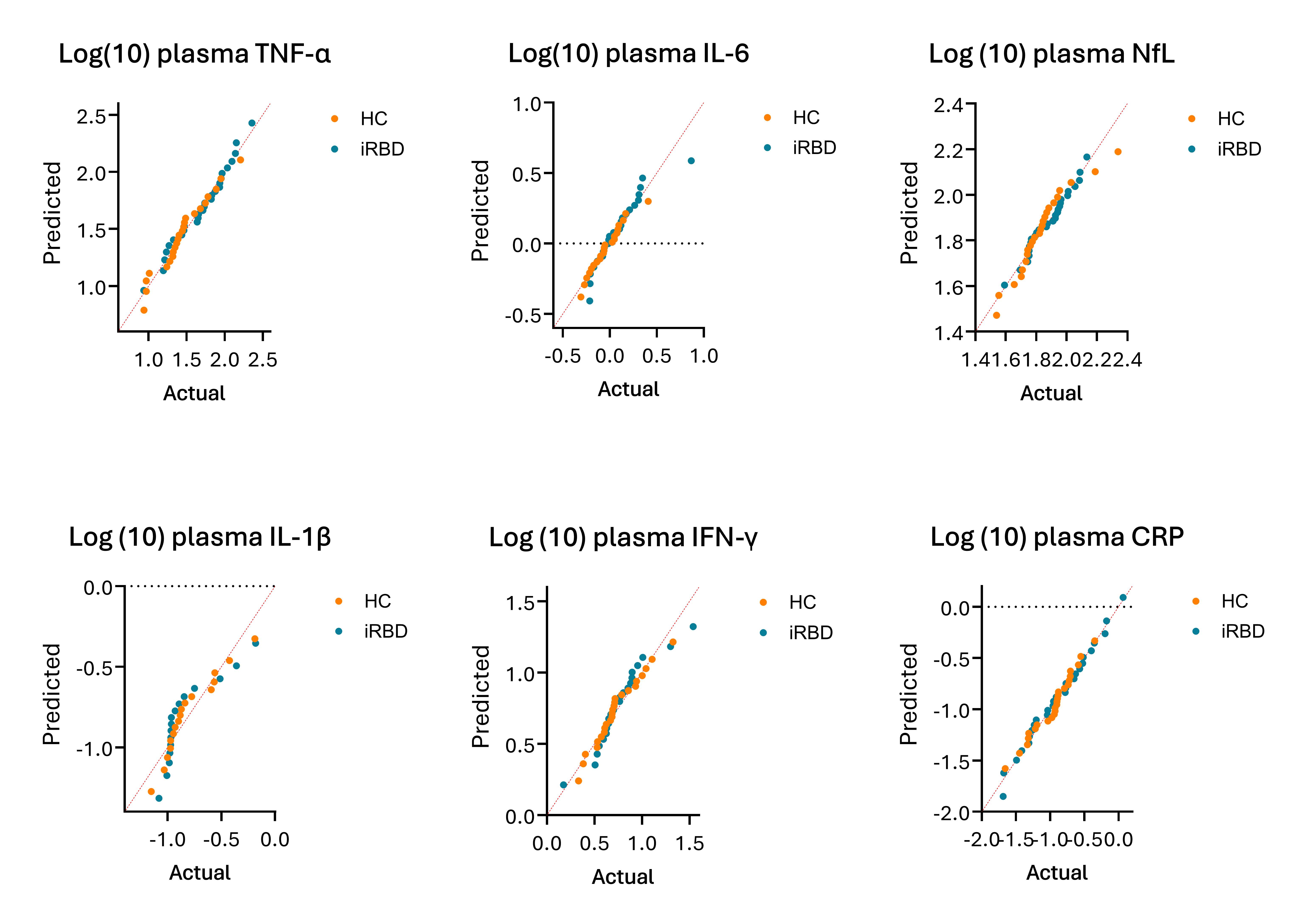

Supplement: Supplementary file 3 — Figure S3. Quantile‐quantile (Q‐Q) plots for log plasma biomarkers. Q‐Q plots for log (base 10) transformed biomarkers are displayed for log (10) plasma TNF‐α, log (10) plasma IL‐6, log (10) NfL, log (10) plasma IL‐1β, log (10) plasma IFN‐ɣ, and log (10) plasma CRP. Q‐Q plots were visually inspected for normality to assess the suitability of the linear model for age and sex adjustment in the case–control biomarker analysis. [file MDC3-12-1961-s004.png]

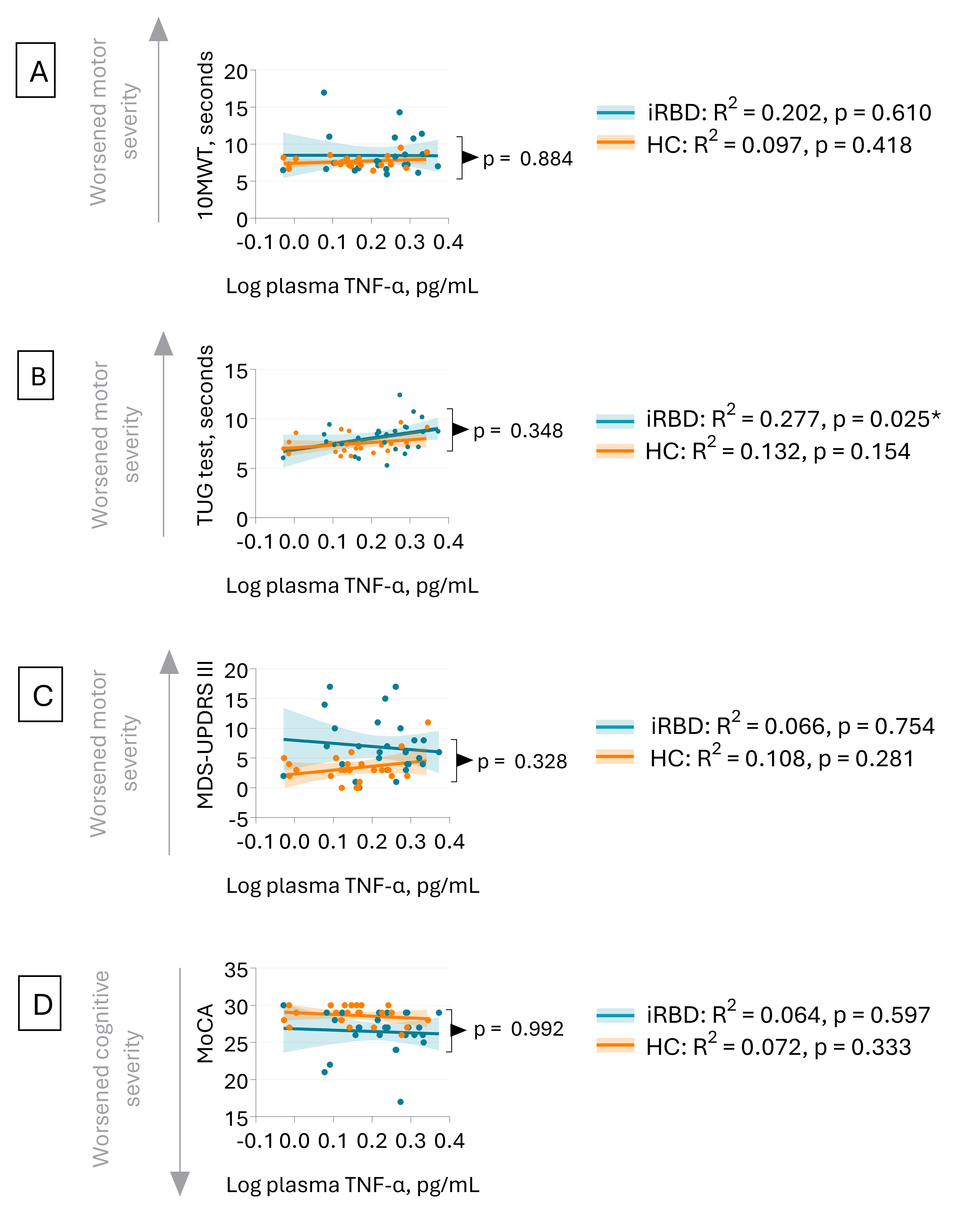

Supplement: Supplementary file 4 — Figure S4. Linear regression of plasma TNF‐α with clinical markers. (A) 10 m walk test (10MWT), (B) Timed Up and Go (TUG) test, (C) MDS‐UPDRS III, and (D) MoCA. Reported p‐values are adjusted for age and sex. [file MDC3-12-1961-s002.png]

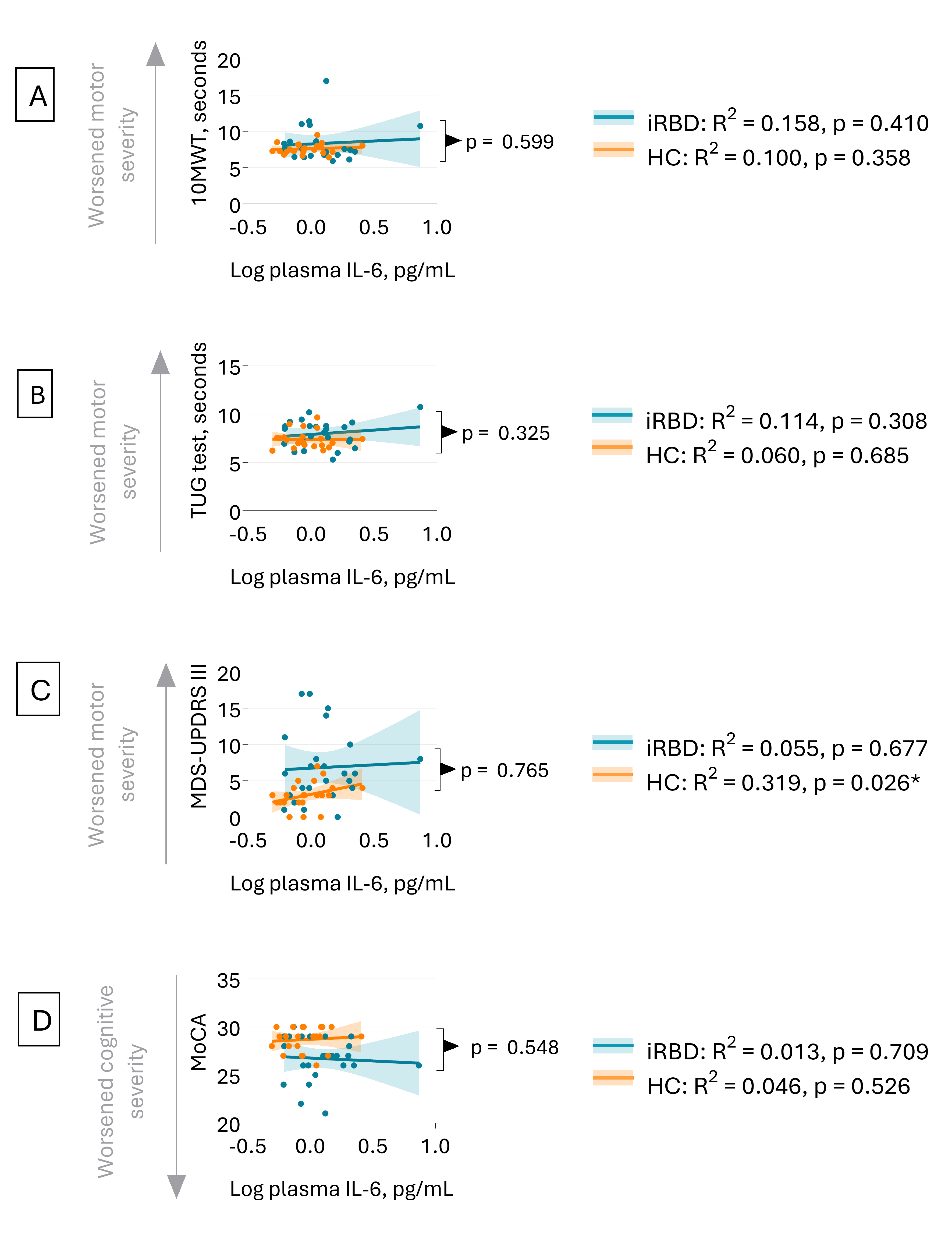

Supplement: Supplementary file 5 — Figure S5. Linear regression of plasma IL‐6 with clinical markers. (A) 10 m walk test (10MWT), (B) Timed Up and Go (TUG) test, (C) MDS‐UPDRS III, and (D) MoCA. Reported p‐values are adjusted for age and sex. [file MDC3-12-1961-s005.png]
