# Supplementary material for: Plasma Neurofilament Light Chain and TNF‐α Correlate with Motor Features in Isolated REM Sleep Behavior Disorder
Source: Mov Disord Clin Pract. 2025 Jul 14;12(11):1961–7. doi: 10.1002/mdc3.70210 (PMC12625127; doi:10.1002/mdc3.70210)
Supplement: Supplementary file 6 — TABLE S1. Intra‐plate NfL assay characteristics. For each NfL plate, the lower limit of detection (LLOD) is reported, along with the average %Coefficient of Variation (CV) of all duplicate plasma samples, the number of plasma samples that fell outside the detection range, and the number of plasma samples with %CVs greater than 25% TABLE S2. Inter‐plate NfL control sample assay characteristics. Control samples were chosen as low, medium, and high NfL plasma samples from first plate run. Reported for the three controls are: the average control NfL value across plates 2–4, the inter‐plate control %Coefficient of Variation (CV) across all four plates, the value of plasma NfL for each control from first plate run, and the average control % recovery for plates 2–4 TABLE S3. Intra‐plate CRP assay characteristics. For each CRP plate, the lower limit of detection (LLOD) is reported, along with the average %Coefficient of Variation (CV) of all duplicate plasma samples, the number of plasma samples that fell outside the detection range, and the number of plasma samples with %CVs greater than 25% TABLE S4. Inter‐plate CRP control sample assay characteristics. Control samples were chosen as low, medium, and high CRP plasma samples from plate 1. Reported for the three controls are: the average control CRP value for plate 2, the inter‐plate control %Coefficient of Variation (CV) across the two plates, the value of plasma CRP for each control from plate 1, and the average control % recovery for plate 2. *Average of CRP control samples from plate 2; plate 1 was used to determine plasma CRP concentration of control samples and for calculation of % recovery TABLE S5. Intra‐plate pro‐inflammatory cytokines (IFN‐ɣ, IL‐1β, IL‐6, and TNF‐α) assay characteristics. For each plate and cytokine, the lower limit of detection is reported, along with the average %Coefficient of Variation (CV) for all duplicate plasma samples run, the number of plasma samples that fell outside the detection range [file MDC3-12-1961-s003.docx]

**RBD manuscript supplementary information**

**e-Methods**

**Plasma analysis**

**Analysis of plasma pro-inflammatory cytokines (MSD V-Plex pro-inflammatory Panel I kit:** **IFN-ɣ, IL-1β, IL-6, TNF-α):** calibrators, controls, and 2-fold diluted plasma samples were added to the 96-well plate and incubated with shaking. The plate was then washed, and antibody detection solution added with incubation and shaking. Finally, the plate was washed again before adding a read-buffer to each well and reading using the MSD QuickPlex instrument. After running all plasma samples with a 2-fold dilution in duplicate on plate 1 and 2, each plasma sample was then run again in singlicate on plate 3 without the 2-fold dilution, as the IL-1β levels were below the limit of detection when run with a 2-fold dilution, resulting in triplicate measurements for all samples.

**Analysis of NfL (MSD R-Plex NfL kit):** firstly, the plate was coated with capture-antibody solution and incubated with shaking overnight. Next, the plate was washed, and calibrators, controls, and 2-fold diluted plasma samples were added to the 96-well plate and incubated with shaking. The plate was then washed again, and detection antibody added to each well with incubation and shaking. Finally, the plate was washed again before adding a read-buffer to each well and reading using the MSD QuickPlex instrument.

**Analysis of CRP (MSD V-Plex CRP kit):** calibrators, controls and 1000-fold diluted plasma samples were added to the 96-well plate and incubated with shaking. The plate was then washed, and detection antibody added to each well with incubation and shaking. Finally, the plate was washed again before adding a read-buffer to each well and reading using the MSD QuickPlex instrument.

**Plasma sample data analysis:** Raw plate data files were downloaded from the MSD Discovery Workbench software, then imported into R Studio, and all plate data from multiple plates and assays combined into one data frame. Any data points that were below the fit curve range or below the detection range were removed, leaving only data points that were in the detection range of each assay. Data was then averaged over the duplicate/triplicate measurements for each biofluid, participant, and assay type. Percent CV was calculated as shown in Equation S1.

|  | $Percent coefficient of variation, \%CV = \frac{Standard deviation}{Average}\times100$ | (Equation S1) |
| --- | --- | --- |

All data were then filtered for percent coefficient of variation (%CVs) less than 25%, and any data points where the %CV of the duplicate/triplicate measurement was > 25% were removed from the analyses. Plasma results are given in the results section as the average of triplicate measurements for all cytokines (IFN-ɣ, IL-6, and TNF-α) except for IL-1β (low circulating levels of IL-1β in plasma meant that the first set of duplicate data, which was diluted 2-fold had a lot of results below the LLOD, meaning that some of the IL-1β data is singlicate data, or sparse), and in duplicate for NfL and CRP.

Inter-plate variability was assessed by reporting the %CVs for controls across all plates run and a %CV of less than 25% was deemed acceptable. Also reported for inter-plate variability was the average % recovery for control samples, with the acceptable range from 80-120%. (Equation S2 shows how the percentage recovery of control samples was calculated.

|  | $Percentage recovery, \% = \frac{Calculated concentration, pg/mL}{Expected concentration, pg/mL} \times100$ | (Equation S2) |
| --- | --- | --- |

Assay reliability and accuracy were checked by reporting the intra-assay characteristics (for each plate): the lower limit of detection (LLOD), the average %CV for all plasma samples, the number of plasma samples with %CVs greater than the cut-off of 25%, and the number of plasma samples that fell outside the detection range of the assays.

**Statistical analysis**

**For demographic and clinical data comparisons:** All data were checked for normality using the Shapiro-Wilk test for normality; all variables were non-normally distributed, therefore the Mann-Whitney U test was performed for all clinical and demographic parameters. Pearson’s chi squared test was used to compare gender between the two groups.

**For biomarker case-control comparisons:** Plasma biomarker data were firstly checked for normality using the Shapiro-Wilk test for normality with a cut-off of p > 0.05 for assumed normality. If data were not normally distributed, the data were log-transformed (base 10) and re-checked for normality using the Shapiro Wilk test. Where raw or log-transformed data were normally distributed, the parametric t-test was performed on the raw/ log-transformed data, respectively. Where neither the raw nor log-transformed data were normally distributed, the non-parametric Mann-Whitney U test was performed on the raw data.

To adjust for age and sex, a linear model (LM) was used. All plasma biomarker data were log transformed (base 10), and the quantile-quantile (Q-Q) plots were inspected visually for normality (Figure S3). Both the un-adjusted LM p-values and adjusted (for age and sex) p-values are reported (Table S8). Finally, to assess effect size, Cohen’s d test was performed, with the following cut-offs used (<0.2: ‘negligible effect’, 0.2-0.5: ‘small effect’, 0.5-0.8: ‘medium effect’ and 0.8-1: ‘large effect’).

**For correlation analysis of plasma biomarkers with clinical markers:** Multiple linear regression analyses were performed on both the raw plasma biomarker (Table S9) and log (base 10) transformed plasma biomarker data (Table S10, Figure 1, Figures S4-S5). Q-Q plots of residuals were visually inspected for normality for each marker to ensure data followed a normal distribution. Linear models were adjusted for age and sex and R^2^ and adjusted p-values reported.

**Tables**

**Table S1 Intra-plate NfL assay characteristics.** For each NfL plate, the lower limit of detection (LLOD) is reported, along with the average %Coefficient of Variation (CV) of all duplicate plasma samples, the number of plasma samples that fell outside the detection range, and the number of plasma samples with %CVs greater than 25%.

|  |  | **Plate 1** | **Plate 2** | **Plate 3** | **Plate 4** |
| --- | --- | --- | --- | --- | --- |
| **NfL** | LLOD, pg/mL | 12.1 | 12.7 | 12.7 | 11.0 |
|  | # plasma samples %CV > 25% * | 0 | 0 | NA | NA |
|  | # plasma samples outside detection range * | 0 | 0 | NA | NA |
|  | Intra-run % CV, plasma samples | 4.89 | 5.01 | NA | NA |

**Table S2 Inter-plate NfL control sample assay characteristics.** Control samples were chosen as low, medium, and high NfL plasma samples from first plate run. Reported for the three controls are: the average control NfL value across plates 2-4, the inter-plate control %Coefficient of Variation (CV) across all four plates, the value of plasma NfL for each control from first plate run, and the average control % recovery for plates 2-4.

| **Plate** | **Control #** | **Average, pg/mL *** | **Inter-plate control %CV (4 plates)** | **Plasma NfL, plate 1, pg/mL** | **Average control % recovery** |
| --- | --- | --- | --- | --- | --- |
| NfL | 1 | 202.4 | 14.4 | 217.7 | 93.0 |
|  | 2 | 108.7 | 2.8 | 107.1 | 101.5 |
|  | 3 | 37.1 | 5.2 | 34.7 | 106.9 |

**Table S3 Intra-plate CRP assay characteristics.** For each CRP plate, the lower limit of detection (LLOD) is reported, along with the average %Coefficient of Variation (CV) of all duplicate plasma samples, the number of plasma samples that fell outside the detection range, and the number of plasma samples with %CVs greater than 25%.

|  |  | **Plate 1** | **Plate 2** |
| --- | --- | --- | --- |
| **CRP** | LLOD, pg/mL | 2.35 | 1.96 |
|  | # plasma samples %CV > 25% * | 0 | 0 |
|  | # plasma samples outside detection range * | 0 | 0 |
|  | Intra-run % CV, plasma samples | 1.53 | 1.17 |

**Table S4 Inter-plate CRP control sample assay characteristics.** Control samples were chosen as low, medium, and high CRP plasma samples from plate 1. Reported for the three controls are: the average control CRP value for plate 2, the inter-plate control %Coefficient of Variation (CV) across the two plates, the value of plasma CRP for each control from plate 1, and the average control % recovery for plate 2. *Average of CRP control samples from plate 2; plate 1 was used to determine plasma CRP concentration of control samples and for calculation of % recovery.

| **Plate** | **Control #** | **Average, mg/dL *** | **Inter-plate control %CV (2 plates)** | **Plasma CRP, plate 1, mg/dL** | **Average control % recovery** |
| --- | --- | --- | --- | --- | --- |
| CRP | 1 | 0.010 | 2.04 | 0.010 | 97.2 |
|  | 2 | 0.153 | 6.03 | 0.166 | 91.8 |
|  | 3 | 1.15 | 1.25 | 1.17 | 98.2 |

**Table S5 Intra-plate pro-inflammatory cytokines (IFN-ɣ, IL-1β, IL-6, and TNF-α) assay characteristics.** For each plate and cytokine, the lower limit of detection is reported, along with the average %Coefficient of Variation (CV) for all duplicate plasma samples run, the number of plasma samples that fell outside the detection range of the assay, and the number of plasma samples with %CVs greater than the cut-off (25%). *Plasma samples run in singlicate.

|  |  | **Plate 1** | **Plate 2** | **Plate 3 *** | **Plate 4** | **Plate 5** |
| --- | --- | --- | --- | --- | --- | --- |
| **IFN-γ** | LLOD, pg/mL | 0.25 | 0.27 | 0.05 | 0.35 | 0.36 |
|  | # plasma samples %CV > 25% | 0 | 0 | NA | NA | NA |
|  | # plasma samples outside detection range | 0 | 0 | 0 | NA | NA |
|  | Intra-run % CV, plasma samples | 5.96 | 6.62 | NA | NA | NA |
| **IL-1β** | LLOD, pg/mL | 0.05 | 0.07 | 0.04 | NA | 0.03 |
|  | # plasma samples %CV > 25% | 8 | 1 | NA | NA | NA |
|  | # plasma samples outside detection range | 14/28 | 27/27 | 10/55 | NA | NA |
|  | Intra-run % CV, plasma samples | 25.54 | 140.98 | NA | NA | NA |
| **IL-6** | LLOD, pg/mL | 0.10 | 0.08 | 0.14 | 0.09 | 0.11 |
|  | # plasma samples %CV > 25% | 0 | 0 | NA | NA | NA |
|  | # plasma samples outside detection range | 0 | 0 | 0 | NA | NA |
|  | Intra-run % CV, plasma samples | 5.67 | 5.64 | NA | NA | NA |
| **TNF-****α** | LLOD, pg/mL | 0.07 | 0.09 | 0.06 | 0.10 | 0.13 |
|  | # plasma samples %CV > 25% | 0 | 0 | NA | NA | NA |
|  | # plasma samples outside detection range | 0 | 0 | 0 | NA | NA |
|  | Intra-run % CV, plasma samples | 4.69 | 5.34 | NA | NA | NA |

**Table S6 Inter-plate pro-inflammatory cytokines (IFN-ɣ, IL-1β, IL-6, and TNF-α) control sample assay characteristics.** For each control sample supplied by Meso Scale Discovery (MSD), the average concentration is reported across 5 plates, the inter-plate control %coefficient of variation (CV) is reported, the expected control concentration from MSD is reported, along with the average control % recovery for the five plates.

| **Plate** | **Control #, pg/mL** | **Average, pg/mL** | **Inter-plate control %CV (5 plates)** | **[MSD Control], pg/mL** | **Average control % recovery** |
| --- | --- | --- | --- | --- | --- |
| **IFN-ɣ plate** | **1** | 614.7 | 4.33 | 608 | 101.1 |
|  | **2** | 168.0 | 1.56 | 156 | 107.7 |
|  | **3** | 48.2 | 5.45 | 41.5 | 116.1 |
| **IL-1β plate** | **1** | 207.7 | 21.01 | 218 | 95.3 |
|  | **2** | 52.6 | 20.74 | 53 | 99.3 |
|  | **3** | 11.8 | 21.45 | 11.2 | 105.1 |
| **IL-6 plate** | **1** | 256.3 | 3.33 | 258 | 99.3 |
|  | **2** | 50.8 | 4.46 | 47.7 | 106.4 |
|  | **3** | 8.8 | 4.73 | 7.5 | 117.1 |
| **TNF-α plate** | **1** | 81.7 | 2.25 | 83 | 98.5 |
|  | **2** | 17.2 | 5.44 | 15.4 | 111.4 |
|  | **3** | 2.1 | 18.79 | 1.88 | 111.4 |

**Table S7 Demographics and clinical data for iRBD patients (n = 27) and controls (n = 25) who were eligible for the study and provided a blood sample.** All data are presented as mean and standard deviation, apart from gender, which is presented as the number of males as a percentage of each group. Pearson’s chi squared test was used to compare gender between the two groups, and the Mann-Whitney U Test to compare between groups for all other data, with a significance of 5%. *p<0.05. SD = standard deviation.

|  | **iRBD** | **HC** | **p-value** |
| --- | --- | --- | --- |
| **Number** | 27 | 25 | NA |
| **Age, mean years (SD)** | 68.41 (8.28) | 69.56 (6.78) | 0.607 |
| **Male, number (%)** | 22 (81.48%) | 21 (84.00%) | 0.810 |
| **Disease duration, mean years since diagnosis (SD)** | 6.78 (2.85) | NA | NA |
| **MDS UPDRS Part III, mean (SD)** | 6.85 (4.70) | 3.16 (2.41) | <0.001* |
| **MoCA, mean (SD)** | 26.44 (2.90) | 28.64 (1.19) | 0.001* |
| **Timed Up and Go (TUG) test, seconds (SD)** | 8.13 (1.56) | 7.42 (0.94) | 0.086 |
| **10m walk test (10MWT), seconds (SD)** | 8.46 (2.70) | 7.65 (0.73) | 0.836 |

**Table S8 Plasma biomarker levels for iRBD patients and healthy controls (HC) adjusted for age and sex.** Plasma biomarker levels are given as mean levels and standard deviations (SD). P-values are given (A) un-adjusted using T-tests or Mann-Whitney U Test as in the main manuscript, (B) un-adjusted using a linear model, and (C) adjusted for age and sex using a linear model. Effect size using Cohen’s d is reported. n = number of participants, _MW_: Mann Whitney U-Test, _T_: T-test, *p < 0.05.

|  | **iRBD** | **HC** | **(A) Un-adjusted p-value** | **(B) LM un-adjusted p-value (log)** | **(C) LM adjusted p-value (age and sex)** | **Effect size**  **(Cohen’s d)** |
| --- | --- | --- | --- | --- | --- | --- |
| **[Plasma TNF-α], pg/mL**  **mean (SD)** | 1.70 (0.352)  *n = 27* | 1.45 (0.326)  *n = 23* | 0.013* _T_ | 0.016* | 0.022* | -0.730 (medium) |
| **[Plasma IL-6], pg/mL**  **mean (SD)** | 1.50 (1.35)  *n = 24* | 0.987 (0.451)  *n = 22* | 0.051 _MW_ | 0.043* | 0.047* | -0.495 (small) |
| **[Plasma NfL], pg/mL**  **mean (SD)** | 80.3 (24.7)  *n = 27* | 74.0 (38.7)  *n = 25* | 0.085 _MW_ | 0.212 | 0.127 | -0.195 (negligible) |
| **[Plasma IL-1β], pg/mL mean (SD)** | 0.181 (0.158)  *n = 16* | 0.190 (0.144)  *n = 17* | 0.533 _MW_ | 0.698 | 0.699 | 0.061 (negligible) |
| **[Plasma IFN-ɣ], pg/mL mean (SD)** | 7.40 (6.96)  *n = 23* | 6.27 (4.22)  *n = 24* | 0.597 _T (log)_ | 0.597 | 0.644 | -0.197 (negligible) |
| **[Plasma CRP], mg/dL**  **mean (SD)** | 0.225 (0.258)  *n = 27* | 0.137 (0.093)  *n = 25* | 0.485 _T (log)_ | 0.491 | 0.484 | -0.444 (small) |

**Table S9 Correlation analyses of plasma NfL, TNF-α, and IL-6 with clinical markers.** Clinical markers analysed were 10-m walk test (10MWT), Timed Up and Go (TUG) test, MDS-UPDRS III, and MoCA. Data are raw (not log transformed). Reported p-values are adjusted for age and sex. *p<0.05.

| **Clinical test** | **Biomarker** | **HC** | | **iRBD** | | **Are slopes significantly different?** |
| --- | --- | --- | --- | --- | --- | --- |
|  |  | **R^2^** | **Adjusted p value** | **R^2^** | **Adjusted p value** |  |
| **10MWT, seconds** | **Plasma NfL** | 0.075 | 0.314 | 0.371 | **0.021*** | **0.001*** |
|  | **Plasma TNF-α** | 0.110 | 0.336 | 0.201 | 0.631 | 0.805 |
|  | **Plasma IL-6** | 0.098 | 0.365 | 0.170 | 0.331 | 0.667 |
| **TUG test, seconds** | **Plasma NfL** | 0.069 | 0.713 | 0.263 | **0.032*** | **0.018*** |
|  | **Plasma TNF-α** | 0.167 | 0.093 | 0.274 | **0.026*** | 0.521 |
|  | **Plasma IL-6** | 0.064 | 0.623 | 0.202 | 0.074 | 0.264 |
| **MDS-UPDRS III** | **Plasma NfL** | 0.060 | 0.374 | 0.122 | 0.212 | **0.040*** |
|  | **Plasma TNF-α** | 0.148 | 0.152 | 0.069 | 0.685 | 0.219 |
|  | **Plasma IL-6** | 0.287 | **0.043*** | 0.052 | 0.750 | 0.676 |
| **MoCA** | **Plasma NfL** | 0.018 | 0.738 | 0.102 | 0.264 | 0.130 |
|  | **Plasma TNF-α** | 0.095 | 0.231 | 0.060 | 0.686 | 0.804 |
|  | **Plasma IL-6** | 0.048 | 0.509 | 0.011 | 0.763 | 0.605 |

**Table S10 Correlation analyses of log plasma NfL, TNF-α, and IL-6 with clinical markers.** Clinical markers analysed were 10-m walk test (10MWT), Timed Up and Go (TUG) test, MDS-UPDRS III, and MoCA. Plasma biomarker data are log transformed. Reported p-values are adjusted for age and sex. *p<0.05.

| **Clinical test** | **Log biomarker** | **HC** | | **iRBD** | | **Are slopes significantly different?** |
| --- | --- | --- | --- | --- | --- | --- |
|  |  | **R^2^** | **Adjusted p value** | **R^2^** | **Adjusted p value** |  |
| **10MWT, seconds** | **Plasma NfL** | 0.152 | 0.181 | 0.355 | **0.034*** | **0.003*** |
|  | **Plasma TNF-α** | 0.097 | 0.418 | 0.202 | 0.610 | 0.884 |
|  | **Plasma IL-6** | 0.100 | 0.358 | 0.158 | 0.410 | 0.599 |
| **TUG, seconds** | **Plasma NfL** | 0.126 | 0.776 | 0.292 | **0.027*** | **0.026*** |
|  | **Plasma TNF-α** | 0.132 | 0.154 | 0.277 | **0.025*** | 0.348 |
|  | **Plasma IL-6** | 0.060 | 0.685 | 0.114 | 0.308 | 0.325 |
| **MDS-UPDRS III** | **Plasma NfL** | 0.085 | 0.472 | 0.197 | 0.145 | **0.042*** |
|  | **Plasma TNF-α** | 0.108 | 0.281 | 0.066 | 0.754 | 0.328 |
|  | **Plasma IL-6** | 0.319 | **0.026*** | 0.055 | 0.677 | 0.765 |
| **MoCA** | **Plasma NfL** | 0.015 | 0.934 | 0.176 | 0.302 | 0.134 |
|  | **Plasma TNF-α** | 0.072 | 0.333 | 0.064 | 0.597 | 0.992 |
|  | **Plasma IL-6** | 0.046 | 0.526 | 0.013 | 0.709 | 0.548 |
